# Effect of a Probiotic Combination on Clinical and Microbiological Oral Parameters in Head and Neck Cancer Patients: A Randomised Clinical Trial

**DOI:** 10.3390/cancers17152459

**Published:** 2025-07-25

**Authors:** Tanya Pereira Riveros, Enric Jané Salas, Alicia Lozano Borbalas, Felipe Rodrigo Aguilera, Teresa Vinuesa Aumedes

**Affiliations:** 1 Laboratory of Molecular Microbiology & Antimicrobials, Department of Pathology and Experimental Therapeutics, Faculty of Medicine, University of Barcelona, 08908 Barcelona, Spain; tanya.pereira@ub.edu (T.P.R.); faguileramunoz@gmail.com (F.R.A.); 2 Department of Odontostomatology, Faculty of Medicine, University of Barcelona, 08908 Barcelona, Spain; enricjanesalas@ub.edu; 3 Department of Radiation Oncology, Catalan Institute of Oncology, L’Hospitalet de Llobregat, 08908 Barcelona, Spain; alozano@iconcologia.net; 4 Instituto de Odontoestomatología, Facultad de Medicina, Universidad Austral de Chile, Valdivia 5090000, Chile

**Keywords:** head and neck cancer, radiotherapy, probiotic combination, oral microbiota, xerostomia, periodontopathogens, randomised clinical trial, Spain

## Abstract

Objective: To evaluate the effect of a probiotic combination on clinical and oral microbiological parameters in patients with head and neck cancer (HNC) undergoing radiotherapy. Materials and Methods: A randomised, double-blind, placebo-controlled clinical trial was conducted with 72 HNC patients who had received radiotherapy within the past year. Participants were randomly assigned to receive either daily probiotic sachets or placebo for 30 days. Salivary parameters—including unstimulated and stimulated flow rates and pH—were evaluated alongside oral microbiota profiles, including total bacterial load and selected periodontopathogens. Assessments were performed at baseline and post-intervention using sialometry, pH analysis, bacterial culture, and quantitative real-time PCR (qPCR). Results: Sixty-one patients completed the study (31 in the probiotic group, 30 in the placebo group). Stimulated salivary flow increased significantly in the probiotic group (*p* = 0.0016), while unstimulated flow improved in both groups (*p* < 0.05). Salivary pH decreased significantly in the probiotic group (*p* = 0.0209); however, no intergroup differences were observed at the end of the intervention (*p* = 0.9839). qPCR showed significant reductions in total bacterial load (*p* = 0.0209) and *Fusobacterium nucleatum* (*p* = 0.0080). Culture confirmed the reduction of *F. nucleatum* (*p* = 0.0026), with a trend towards significance for total cultivable bacterial count (*p* = 0.0502). Conclusions: Daily supplementation with a probiotic combination may serve as a practical and well-tolerated adjunctive measure in clinical settings to improve salivary function and reduce key oral pathogens, particularly *Fusobacterium nucleatum*, in patients undergoing or recovering from radiotherapy for head and neck cancer. These findings support its potential integration into routine supportive care protocols to mitigate xerostomia and oral dysbiosis in this population.

## Simple Summary

Patients with head and neck cancer undergoing radiotherapy frequently experience xerostomia and disturbances in the oral bacterial balance, which can significantly impair their quality of life. This study investigated whether a 30-day daily intake of a probiotic combination could enhance salivary function and reduce disease-associated oral bacteria. Participants were randomly assigned to receive either probiotics or a placebo. By the end of the intervention, those in the probiotic group showed notable improvements in stimulated salivary flow and a reduction in overall bacterial counts, particularly *Fusobacterium nucleatum*. These results suggest that probiotics may represent a safe and effective approach to prevent or mitigate radiotherapy-induced oral complications. This study highlights the potential role of probiotics as a simple and supportive measure to improve oral health and overall well-being during cancer treatment recovery.

## 1. Introduction

Head and neck cancer (HNC) comprises a heterogeneous group of malignant neoplasms affecting the upper aerodigestive tract, including the oral cavity, pharynx (nasopharynx, oropharynx, and hypopharynx), larynx, salivary glands, and nasal cavity [1]. These tumours encompass diverse histological types with distinct clinical, prognostic, and molecular characteristics [2]. Among them, the most prevalent subtype is head and neck squamous cell carcinoma (HNSCC), which accounts for approximately 90% of all HNC cases. HNSCC arises from the squamous epithelium lining most of these anatomical sites and represents the primary focus of current clinical and epidemiological research [3]. Collectively, head and neck cancers rank as the seventh most common type of cancer worldwide, underscoring their global oncological relevance [4].

Radiotherapy in patients with head and neck cancer (HNC) is frequently associated with significant adverse effects [5]. The most common complications include oral mucositis (OM), dysgeusia, salivary gland dysfunction, infections, tissue necrosis, and periodontal disease [6]. It is estimated that radiotherapy-induced oral mucositis affects 80–100% of patients with HNC, whilst xerostomia may persist in up to 90% of cases, even months or years following treatment [7,8]. Exposure to ionising radiation damages the glandular parenchyma, compromising both the quantity and quality of salivary flow. Clinically, this manifests as xerostomia, often accompanied by alterations in the biochemical composition, pH, and viscosity of saliva [9].

These changes not only impair patients’ quality of life but also create an oral environment conducive to microbial dysbiosis [10]. Several studies have demonstrated that radiotherapy in HNC patients induces substantial alterations in the oral microbial ecology, promoting a dysbiotic state characterised by shifts in bacterial diversity and relative abundance [11,12].

Of particular concern are periodontopathogenic bacteria, including *Porphyromonas gingivalis*, *Tannerella forsythia*, *Campylobacter rectus*, *Aggregatibacter actinomycetemcomitans*, and *Fusobacterium nucleatum*, which are strongly associated with chronic inflammatory conditions and have been implicated in the progression of systemic diseases [13,14,15]. These findings underscore the need for preventive and therapeutic strategies aimed at modulating the oral microbiota.

In this context, probiotics—live microorganisms that, when administered in adequate amounts, confer health benefits to the host—have emerged as a promising approach for restoring oral eubiosis [16,17]. The most commonly used probiotics belong to the genera *Lactobacillus*, *Bifidobacterium*, *Streptococcus*, and *Saccharomyces*. Among these, species such as *Lactobacillus rhamnosus*, *L. reuteri*, *L. acidophilus*, *L. casei*, and *L. plantarum*, as well as *Bifidobacterium breve*, *B. longum*, and *B. infantis*, are frequently employed in clinical studies owing to their well-documented safety profile [18]. Their modulatory effects on the microbiota are attributed to their ability to compete with pathogens for nutrients and adhesion sites, as well as their production of antimicrobial compounds such as bacteriocins and organic acids [19]. Various strains, including *Lactobacillus reuteri*, *Lactobacillus rhamnosus*, and *Bifidobacterium* spp., have demonstrated the capacity to inhibit the growth and virulence of periodontopathogenic bacteria such as *P. gingivalis*, *A. actinomycetemcomitans*, *T. forsythia*, and *F. nucleatum* [20,21,22,23].

Beyond oral health, probiotics have demonstrated beneficial effects in other chronic inflammatory conditions. In allergic rhinitis, a recent randomised, double-blind, placebo-controlled trial showed that a 90-day supplementation with a probiotic–prebiotic combination significantly reduced symptom severity and modulated inflammatory markers such as TNF-α, IFN-γ, and IL-17. These effects were linked to alterations in gut microbiota composition and increased production of short-chain fatty acids, underscoring the immunomodulatory potential of probiotics [24]. Probiotic supplementation has also been investigated as an adjunctive therapy in metabolic diseases such as type 2 diabetes mellitus (T2DM). A recent meta-analysis of 30 randomised clinical trials involving over 1800 patients reported significant improvements in glycaemic control, including reductions in fasting glucose, insulin levels, HbA1c, and HOMA-IR indices [25]. Furthermore, probiotics have shown promise in chronic immune-mediated skin disorders such as psoriasis. In a 12-week clinical trial, patients receiving topical therapy alongside probiotic–prebiotic supplementation exhibited improvements in disease severity, quality of life, and systemic inflammatory markers, along with favourable modulation of the gut microbiota towards an anti-inflammatory profile [26]. Collectively, these findings highlight the expanding role of probiotics as adjunctive tools in managing systemic inflammation and microbiota dysregulation across diverse clinical settings, thereby supporting their potential relevance in oncology-related supportive care.

Therefore, this study aimed to investigate the impact of a 30-day oral probiotic intervention in patients with head and neck cancer who had previously undergone radiotherapy. The primary objectives were to evaluate changes in salivary flow parameters (unstimulated and stimulated) and in oral bacterial loads—both total and specific species, including *P. gingivalis*, *F. nucleatum*, *A. actinomycetemcomitans*, *C. rectus*, and *T. forsythia*. Secondary objectives included assessing salivary pH, adherence to treatment, and the occurrence of adverse events. Accordingly, the study addressed the following research questions:

1. Can probiotic supplementation enhance salivary gland function in patients after radiotherapy?
2. Does it reduce overall bacterial burden and the prevalence of periodontopathogens in the oral cavity?
3. Is it a safe and well-tolerated adjuvant option in the context of supportive cancer care?

## 2. Materials and Methods

### 2.1. Study Design

A randomised, double-blind, placebo-controlled clinical trial was conducted between September 2022 and December 2024, with a 30-day clinical follow-up period.

### 2.2. Participants

Participants were patients with head and neck cancer treated at the Radiotherapy Service of the Catalan Institute of Oncology (ICO) and the Dental Hospital of the University of Barcelona (HOUB).

A total of 180 patients were assessed for eligibility. Of these, 72 met the inclusion and exclusion criteria and were randomly allocated to either the probiotic or placebo group in a 1:1 ratio. The study was approved by the HOUB Clinical Research Ethics Committee (approval code: 22/2021) in July 2021 and was registered at ClinicalTrials.gov (Identifier: NCT06122636) in November 2023. All procedures were conducted in accordance with the Declaration of Helsinki (World Medical Association, 2017).

### 2.3. Inclusion Criteria

- Adults aged over 18 years.
- Histological diagnosis of head and neck cancer.
- Radiotherapy completed within the previous year.
- Presence of at least six teeth in the oral cavity.
- Provided written informed consent to participate in the study.

### 2.4. Exclusion Criteria

- Declined to participate.
- Presence of osteonecrosis.
- Inability to take oral medication.
- Known allergy to probiotics.
- Active antibiotic treatment or use of antibiotics within the previous 30 days.

### 2.5. Outcomes

#### 2.5.1. Primary Outcomes

- Change in unstimulated and stimulated salivary flow (mL/min).
- Change in total and specific bacterial loads (*P. gingivalis*, *F. nucleatum*, *A. actinomycetemcomitans*, *C. rectus*, and *T. forsythia*) assessed by culture and qPCR.

#### 2.5.2. Secondary Outcomes

- Change in salivary pH.
- Adherence to treatment and adverse events, evaluated through a post-intervention survey.

### 2.6. Sample Size Calculation

The sample size was calculated using the G*Power, version 3.1.9.7 (Heinrich Heine University Düsseldorf, Düsseldorf, Germany) programme, following the recommendations of Sharma et al. (2012) [27]. The initial calculation indicated a requirement of 126 patients (63 per group). Considering that the Catalan Institute of Oncology (ICO) treats between 150 and 180 patients with head and neck cancer annually, a finite population correction factor was applied, adjusting the final sample size to 68 patients (34 per group), with a 95% confidence level and 80% statistical power.

### 2.7. Randomisation and Blinding

The randomisation sequence was generated by an independent researcher before the study commenced, using Microsoft Excel (Office 2019). A restricted allocation method with a 1:1 ratio was applied to assign participants to either the probiotic or placebo group. This researcher was not involved in recruitment, sample collection, or data analysis and was solely responsible for maintaining allocation concealment.

Both participants and the principal investigator were blinded to treatment allocation. Blinding was ensured through the use of indistinguishable sachets, prepared by ITF Research Pharma SLU (Madrid, Spain). The company produced both the active product and the placebo, ensuring identical packaging, size, shape, colour, and taste, with the placebo differing only by the absence of the active ingredient.

### 2.8. Intervention

The probiotic group received sachets containing fructooligosaccharides (990 mg), vitamin D_3_ (5 µg), and probiotic strains (*Lacticaseibacillus rhamnosus* GG 1 × 10^10^ CFU; *L. casei*; *Lactobacillus acidophilus*; *L. delbrueckii* subsp. *bulgaricus*; *Bifidobacterium infantis*; *B. brevis*; and *Streptococcus thermophilus* 1 × 10^9^ CFU). The placebo group received sachets containing inactive excipients.

Participants were instructed to dissolve one sachet in a glass of water (250 mL) at room temperature, rinse their mouths for 2–3 min, and then swallow the solution once daily after brushing their teeth without toothpaste. The intervention period lasted 30 days.

### 2.9. Clinical Procedures

Two clinical visits were scheduled: the first for baseline assessment and the second at the end of the intervention, within 15 days following treatment completion.

The following assessments were conducted:

#### 2.9.1. Sialometry (Unstimulated and Stimulated Salivary Flow)

- Unstimulated Salivary Flow:

The patient was seated with the head tilted forward, and saliva was collected for 5 min into a graduated tube. Reference values: >0.25 mL/min (normal, code 0), 0.10–0.25 mL/min (low, code 1), and <0.10 mL/min (very low, code 2).

- Stimulated Salivary Flow:

The patient chewed a piece of paraffin, and saliva was collected for 5 min into a graduated tube. Reference values: >1.0 mL/min (normal, code 0), 0.70–1.0 mL/min (low, code 1), and <0.70 mL/min (very low, code 2).

#### 2.9.2. Salivary pH Measurement

Salivary pH was determined in the unstimulated saliva sample using FILTERLAB ^®^ indicator strips and a colour scale ranging from 1 to 14.

#### 2.9.3. Sampling for Microbiological Analysis

Prior to sialometry, samples of gingival crevicular fluid (GCF) were collected using 10 sterile endodontic paper points (size 30, taper 2%; Maillefer, Ecublens, Switzerland), with one paper point placed per quadrant when possible. Five paper points were transferred to tubes with Reduced Transport Fluid (RTF) for bacterial culture (processed in less than 24 h), while the remaining five were stored at –20 °C for subsequent DNA extraction and quantitative real-time PCR (qPCR) analysis.

At the end of the 30-day intervention, all clinical and microbiological assessments were repeated. Additionally, a survey was administered to evaluate treatment adherence and the presence of any co-interventions during the follow-up period.

### 2.10. Oral Microbiological Evaluation

Microbiological evaluation included the quantification of total bacterial counts and specific periodontopathogens: *Porphyromonas gingivalis*, *Fusobacterium nucleatum*, *Aggregatibacter actinomycetemcomitans*, *Campylobacter rectus*, and *Tannerella forsythia*. Samples were analysed before and after the intervention using both microbiological culture (CFU/mL) and quantitative polymerase chain reaction (qPCR).

#### 2.10.1. Standard Bacterial Culture

Selective culture media were used for bacterial growth. *P. gingivalis* and *F. nucleatum* were cultured on fastidious anaerobic agar (FAA) supplemented with 5% horse blood, while *A. actinomycetemcomitans* and *C. rectus* were cultured in brain heart infusion (BHI) medium enriched with 5 g/L yeast extract, 1.5 g/L sodium fumarate, 1 g/L sodium formate, and 1.8 mg/L vancomycin.

Incubation was performed at 37 °C under anaerobic conditions (Don Withley DG 250 chamber, Don Whitley Scientific, Bingley, UK, 10% CO_2_, 10% H_2_, and 80% N_2_) or under aerobic conditions with 5% CO_2_ for 7 to 15 days. Bacterial identification was based on colony counting, morphological characteristics, and catalase, oxidase, indole, and beta-galactosidase biochemical tests, as well as the BANA test (enzymatic breakdown of N-benzoyl-dl-arginine-2-napthylamide), and when needed using RapID^TM^ ANA II/NH galleries (Thermo Fisher Scientific, Waltham, MA, USA). In cases of uncertain identification, MALDI-TOF mass spectrometry was employed.

#### 2.10.2. Real-Time Quantitative PCR (qPCR)

DNA extraction was performed using the QIAamp DNA Mini Kit (QIAGEN, Valencia, CA, USA), for the preparation of standard curves and the MolYsis Complete5 kit (Molzym GmbH & co. KG, Bremen, Germany) for clinical samples, following the manufacturers’ protocols. DNA concentrations were measured spectrophotometrically using a NanoDrop One system (Thermo Scientific, Waltham, MA, USA).

Quantification of bacterial species was conducted using the TaqMan probe-based qPCR system. Reactions were performed in a final volume of 10 μL using qPCR Master Mix (Sigma-Aldrich, St. Louis, MO, USA) and run on an FQD-48A Bioer thermocycler (Hangzhou, China). The cycling conditions included an initial denaturation at 95 °C for 10 min, followed by 40 cycles of denaturation at 95 °C for 15 s and annealing/extension at species-specific temperatures for 1 min.

Species-specific primers and hydrolysis probes (5′ [6FAM]-labelled, 3′ [TAM]-quenched; Sigma-Aldrich) targeting the 16S rRNA gene of total bacteria and selected periodontopathogens were used. The bacterial DNA copy number in each sample was calculated using standard curves generated from known concentrations, as previously described [28,29]. The detailed sequences of primers, probes, and corresponding amplification temperatures are provided in Supplementary Table S1.

### 2.11. Statistical Analysis

- All statistical analyses were performed using Python (v3.9) with SciPy and Pandas libraries. The Shapiro–Wilk test was used to assess the normality of continuous variables. As most data were non-normally distributed, non-parametric tests were applied throughout.
- For paired data (e.g., baseline vs. post-intervention within the same group), the Wilcoxon signed-rank test was used. Between-group comparisons (probiotic vs. placebo) were evaluated using the Mann–Whitney U test. Associations between salivary and microbiological variables were explored using Spearman’s rank correlation coefficient.
- All tests were two-tailed, and a significance level of *p* < 0.05 was adopted. Where appropriate, results are presented with 95% confidence intervals. Statistical outputs were interpreted in the context of both clinical and biological relevance.

## 3. Results

### 3.1. Participation and Baseline Characteristics

**Figure 1:**
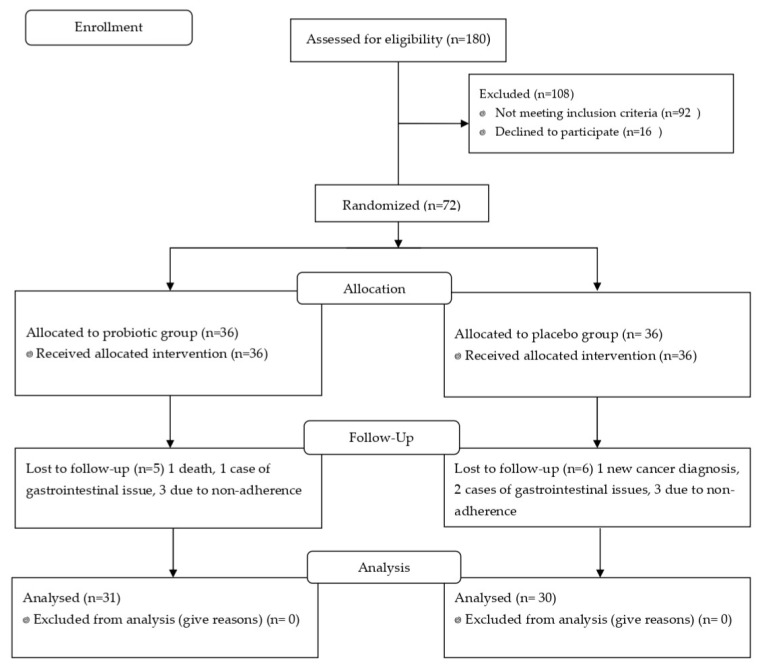
CONSORT flow diagram of patient recruitment and follow-up.

Of the 180 patients assessed for eligibility, 72 met the inclusion and exclusion criteria, provided written informed consent, and were randomly allocated to either the probiotic group (*n* = 36) or the placebo group (*n* = 36). Eleven participants did not complete the study: one died during treatment, one received a new cancer diagnosis, three experienced gastrointestinal discomfort (diarrhoea), and six withdrew due to non-adherence to the intervention protocol. The final analysis included 61 patients: 31 in the probiotic group and 30 in the placebo group. Figure 1, CONSORT.

Demographic and clinical characteristics of the participants are presented in Table 1. Both groups were comparable in terms of age, sex, tumour site, comorbidities, oncological treatment (radiotherapy, chemotherapy, and surgery), smoking status, alcohol consumption, and time since completion of radiotherapy.

The most common tumour sites were the oropharynx and oral cavity. All patients received intensity-modulated radiotherapy (IMRT) with curative intent, with a total dose ranging from 60 to 70 Gy, administered in daily fractions of 2 Gy over 30 to 33 sessions. More than half of the participants underwent chemotherapy, predominantly with cisplatin. Hypertension and diabetes mellitus were the most frequent comorbidities. No statistically significant differences were observed between groups, supporting appropriate baseline comparability.

### 3.2. Salivary Parameters

At baseline, the majority of participants exhibited reduced unstimulated salivary flow, confirming residual salivary dysfunction prior to the intervention. In the placebo group, 80.0% of patients had low flow (0.25–1.0 mL/min), and 20.0% had very low flow (<0.25 mL/min). In the probiotic group, 77.4% had low flow, and 22.6% had very low flow. No statistically significant differences were observed between groups in the distribution of salivary flow categories at baseline (*p* = 0.829, Fisher’s exact test), indicating comparable severity of hyposalivation at study entry.

Changes in salivary flow and pH are summarised in Table 2. Unstimulated salivary flow increased significantly in both groups following the intervention (probiotic group: *p* = 0.0253, *z* = −4.860; placebo group: *p* = 0.0339, *z* = −4.690).

For stimulated salivary flow, only the probiotic group demonstrated a significant improvement after treatment (*p* = 0.0016, *z* = −4.860), while no significant changes were observed in the placebo group (*p* = 0.7055). The comparison between groups also favoured the probiotic group (*p* = 0.0141).

Salivary pH decreased significantly following the intervention in the probiotic group (*p* = 0.0209, *z* = −4.605), indicating a trend towards increased acidity, while no significant changes were observed in the placebo group (*p* = 0.4054). However, no significant intergroup differences were found in post-treatment pH values (*p* = 0.9839). Overall, the probiotic group exhibited clinical improvements as evidenced by an increase in salivary flow, although this was accompanied by a tendency towards acidification of the salivary environment.

### 3.3. Microbiological Analysis

In the probiotic group, qPCR analysis revealed a significant reduction in total bacterial load (*p* = 0.0209, *z* = −2.293) and a significant decrease in *F. nucleatum* levels (*p* = 0.0080, *z* = −2.606). Culture methods confirmed a significant reduction in *F. nucleatum* (*p* = 0.0026, *z* = −2.920), while the reduction in total bacterial count approached statistical significance (*p* = 0.0502, *z* = −1.969) (Table 3).

In the placebo group, no significant differences were observed for any of the variables assessed, either by culture or qPCR.

Post-treatment between-group comparisons showed significant differences favouring the probiotic group in total bacterial load (*p* = 0.0091) and *F. nucleatum* levels (*p* = 0.0019). No significant between-group differences were found for *C. rectus*, *T. forsythia*, *P. gingivalis*, or *A. actinomycetemcomitans*.

### 3.4. Bacterial Detection Frequency

Based on the total patient cohort, *F. nucleatum* demonstrated the highest prevalence, being detected in 96.7% of subjects. This was followed by *C. rectus* (63.9%), *T. forsythia* (56.7%), and *P. gingivalis* (37.7%). *A. actinomycetemcomitans* exhibited the lowest prevalence, with detection in only 1.6% of the cohort.

The distribution of bacterial presence was homogeneous between sexes; however, a higher detection frequency of *F. nucleatum* and *C. rectus* was observed among male participants within the 60–69 years age group, which also presented the highest total bacterial load.

### 3.5. Correlation Between Microbiological Methods

Spearman’s correlation analysis demonstrated a strong association between the results obtained by culture and qPCR for total bacterial counts (probiotic group: *r* = 0.886; placebo group: *r* = 0.765; *p* < 0.001), and an almost perfect correlation for *P. gingivalis* in both groups (*r* > 0.99). High positive correlations were also observed for *F. nucleatum* and *C. rectus*, confirming the consistency between the two microbiological analysis methods (Table 4).

### 3.6. Tolerability and Adverse Events

The intervention was well-tolerated by most participants. Only three patients (4.9%) reported mild episodes of diarrhoea, which led to treatment discontinuation in these cases. No serious adverse events were recorded.

Additionally, some participants in the probiotic group reported subjective improvements in gastrointestinal function following the intervention, including a sensation of abdominal comfort and improved bowel movements.

## 4. Discussion

Given the oncological profile of our cohort—characterised by high-dose IMRT and a substantial proportion of patients receiving chemotherapy—participants were particularly vulnerable to radiation-induced oral toxicities, including xerostomia, mucosal atrophy, and microbial dysbiosis. The presence of common comorbidities such as hypertension and diabetes may have further impaired mucosal repair and immune function. Collectively, these clinical risk factors highlight the importance of exploring adjuvant strategies, such as probiotic supplementation, to mitigate the adverse effects of cancer therapy on the oral environment [30,31,32].

From a clinical perspective, a general improvement in salivary function was observed. Although both groups exhibited significant increases in unstimulated salivary flow following the intervention, the absence of significant between-group differences prevents the attribution of this effect solely to probiotic use. In contrast, stimulated salivary flow showed more consistent improvements in favour of the probiotic group. However, a significant decrease in salivary pH was observed in this group, indicating a shift towards a more acidic environment. These findings suggest that probiotic supplementation may enhance the secretory function of the salivary glands in irradiated patients, although it may not support the maintenance of a neutral oral pH.

While the reduction in salivary pH observed in the probiotic group raises potential concerns regarding cariogenic risk, the magnitude of this change was modest and remained within physiological limits. Moreover, the probiotic formulation administered included *Lactobacillus rhamnosus*, *Lactobacillus acidophilus*, and *Bifidobacterium infantis*—species which, although capable of producing lactic acid, demonstrate limited in vitro adhesion to enamel surfaces and significantly lower biofilm-forming capacity when compared to *Streptococcus mutans* [33,34]. The mild acidification may be partially explained by the fermentation of fructooligosaccharides (FOSs) included in the formulation, which can lead to the production of short-chain fatty acids such as acetate, transiently reducing pH in the oral environment [35]. However, current evidence does not support a direct association between FOS-induced acidification and increased cariogenic potential in the absence of key initiators such as *S. mutans* [36]. Although few clinical trials have examined this topic in cancer populations, our results are consistent with those of Sanghvi et al. (2018) [37], who reported a significant increase in salivary flow after administering a combination of *Lactobacillus rhamnosus* GG-HS111, *L. acidophilus* HS101, and *Bifidobacterium bifidum* in edentulous adults, although without significant changes in pH. These findings support the potential of probiotics as modulators of the oral environment and suggest their application in HNC patients who develop xerostomia secondary to antineoplastic treatment.

Regarding the oral microbiota, our study demonstrated a significant reduction in total bacterial load and *F. nucleatum* levels in the probiotic group, as assessed by both qPCR and culture methods. This finding is particularly relevant given the well-established role of *F. nucleatum* in the pathogenesis of periodontitis [38], as well as its involvement in inflammatory processes, immunomodulation, and carcinogenesis [39,40]. *F. nucleatum* has been shown to promote tumour progression by activating oncogenic pathways such as Wnt/β-catenin and by suppressing host immune responses [41,42]. Tang et al. (2016) [43] demonstrated that *F. nucleatum* induces proinflammatory cytokine production and reactive oxygen species in Caco-2 cells, supporting its role in inflammation and potential mechanisms of carcinogenesis. Subsequent studies have confirmed the high prevalence of *F. nucleatum* in colorectal cancer tissues, associating its presence with tumour progression and metastasis [44,45].

The reduction in *F. nucleatum* observed in this study suggests that specific probiotic combinations may modulate clinically relevant bacterial species. In a pilot study, Vesty et al. (2020) [46] evaluated the effect of *Streptococcus salivarius* M18 in patients undergoing radiotherapy for HNC, reporting a negative interaction between *S. salivarius* and *F. nucleatum* within microbial networks, although no changes in relative abundance were detected. Similarly, Alanzi et al. (2018) [47] demonstrated that supplementation with *Lactobacillus rhamnosus* and *Bifidobacterium lactis* over four weeks significantly reduced *F. nucleatum* levels in saliva and dental plaque in a non-oncological population.

These findings support the hypothesis that certain probiotic strains may modulate the presence of oral pathogens either through competitive exclusion or the production of antimicrobial metabolites [48].

In our study, the detection frequency in the studied population of *F. nucleatum* was (96.7%), making it the most prevalent bacteria identified, followed by *C. rectus* (63.9%), *T. forsythia* (56.7%), and *P. gingivalis* (37.7%). The distribution pattern indicated a higher bacterial load among male participants aged 60–69 years, which also represented the most prevalent age group in the cohort. The minimal presence of *A. actinomycetemcomitans* (1.6%) aligns with previous studies reporting its lower prevalence of oral microbiota in patients affected by radiotherapy [49].

Correlation analysis between microbiological methods demonstrated a strong positive association, particularly for *P. gingivalis* (*r* > 0.99) and total bacterial counts (*r* > 0.88), reinforcing the validity and complementarity of both culture and qPCR techniques in clinical microbiological research.

### 4.1. Clinical Implications

The findings of this study indicate that short-term probiotic supplementation may offer clinically meaningful benefits for patients with head and neck cancer (HNC) who have undergone radiotherapy, particularly by enhancing salivary flow and modulating the oral microbiota. These results are especially relevant given the high prevalence of radiation-induced xerostomia and oral dysbiosis in this population, both of which are associated with impaired oral function, nutritional compromise, and reduced quality of life [50,51].

Clinically, probiotics represent a non-invasive, safe, and well-tolerated adjuvant approach that may support the restoration of oral eubiosis and the maintenance of mucosal integrity in cancer survivors [52].

Their beneficial role is supported by accumulating evidence from other chronic and immune-mediated inflammatory conditions. For instance, randomised controlled trials and meta-analyses have shown that probiotics can reduce symptom severity and systemic inflammation in allergic rhinitis, improve glycaemic control in type 2 diabetes mellitus, and modulate immune responses in psoriasis [53,54,55].

In the context of oncology, a growing body of evidence has explored the use of probiotics in patients with head and neck cancer or those undergoing cancer therapies. For example, Sharma et al. (2012) conducted a randomised clinical trial using *Lactobacillus brevis* CD2 tablets in patients with HNC receiving chemoradiotherapy and reported a significant reduction in the incidence and severity of oral mucositis [27]. Similarly, Doppalapudi et al. (2020) demonstrated that a multi-strain probiotic formulation—including *Lactobacillus acidophilus*, *L. rhamnosus*, and *Bifidobacterium bifidum*—reduced *Candida* colonisation and improved oral comfort in patients undergoing radiotherapy for oral cancer [56]. In another pilot study, Vesty et al. (2020) evaluated the use of *Streptococcus salivarius* M18 in HNC patients post-radiotherapy and observed ecological shifts in the oral microbiota associated with reduced microbial pathogenicity [46]. Collectively, these findings support the integration of probiotics into multidisciplinary supportive care protocols aimed at preserving oral health and promoting overall well-being in cancer survivors.

### 4.2. Study Limitations

Although the results of this study are encouraging, certain limitations should be acknowledged in order to contextualise their interpretation. The sample size, while adequate to detect statistically significant effects on the primary outcomes, was relatively modest and drawn from a single clinical setting. This may limit the generalisability of the findings to broader populations with differing demographic or clinical characteristics. The intervention period was limited to 30 days, which may not fully capture the long-term effects of probiotic supplementation on oral health or microbiota composition. Nonetheless, the clinical and microbiological improvements observed during this timeframe suggest a relevant biological response.

Overall, the study’s methodology and the consistency of results across multiple outcome measures support strong internal validity. Future research involving multicentre cohorts, extended follow-up periods, and complementary immunological markers could help confirm these findings and strengthen their applicability across diverse patient populations.

## 5. Conclusions

The results of this study suggest that oral probiotic supplementation is a safe and well-tolerated adjuvant strategy with the potential to support salivary gland function and modulate the oral microbiota in patients with head and neck cancer following radiotherapy. Notably, the intervention was associated with a significant increase in stimulated salivary flow and a reduction in overall bacterial load, including a marked decrease in *Fusobacterium nucleatum*, a pathogen linked to oral dysbiosis and cancer-related inflammation.

These findings underscore the clinical relevance of exploring probiotics as part of supportive care protocols aimed at mitigating radiation-induced oral complications such as xerostomia and microbial imbalance.

Although the relatively short intervention period, the concurrent systemic therapies, differences between groups, and single-centre design limit the generalisability of the results, the consistency and biological plausibility of the observed effects support further investigation. Future multicentre trials with extended follow-up and additional outcome measures—including immunological and quality of life assessments—are warranted to validate and expand upon these findings.

## Figures and Tables

**Table 1:** Distribution of patients according to treatment group, gender, and age range.

Variable	Placebo (*n* = 30)	Probiotic (*n* = 31)	Total (*n* = 61)
Age, mean (SD), years	57.9 (14.6)	60.1 (12.0)	59.0 (13.2)
Sex, n (%)			
- Male	19 (63.3%)	19 (61.3%)	38 (62.3%)
- Female	11 (36.7%)	12 (38.7%)	23 (37.7%)
Tumour location, n (%):			
- Hypopharynx	0 (0.0%)	1 (3.2%)	1 (1.6%)
- Oral cavity	10 (33.3%)	6 (19.4%)	16 (26.2%)
- Oropharynx	10 (33.3%)	14 (45.2%)	24 (39.3%)
- Larynx	7 (23.3%)	2 (6.5%)	9 (14.8%)
- Nasopharynx	2 (6.7%)	6 (19.4%),	8(13.1%)
- Salivary gland	0 (0.0%)	1 (3.2%)	1 (1.6%)
- Unknown primary (neck)	0 (0.0%)	1 (3.2%)	1 (1.6%)
Adjuvant radiotherapy, n (%):	100%	100%	100%
RT dose, mean (SD), Gy	65.8 (5.4)	67.2 (3.9)	66.5 (4.7)
RT sessions, median (range)	33 (25–35)	33 (30–35)	33 (25–35)
Time since RT, mean (SD), months	2.77 ± 1.99	3.52 ± 2.53	
Chemotherapy, n (%):			
*-* Received QT	19 (63.3%)	16 (51.6%)	35 (36.5%)
QT regimen:			
- Cisplatin	16 (53.3%)	7 (22.6%)	23 (24.0%)
- Cetuximab	0 (0.0%)	1 (3.2%)	1 (1.0%)
- Other	3 (10.0%)	8 (25.8%)	11 (11.5%)
Surgery, n (%)	14 (46.7%)	12 (38.7%)	26 (27.1%)
Comorbidities, n (%)			
- Diabetes mellitus	6 (20.0%)	7 (22.6%)	13 (13.5%)
- Hypertension	11 (36.7%)	12 (38.7%)	23 (24.0%)
- Cardiovascular disease	3 (10.0%)	1 (3.2%)	4 (4.2%)
- Pulmonary disease	1 (3.3%)	2 (6.5%)	3 (3.1%)
- Thyroid disease	1 (3.3%)	2 (6.5%)	3 (3.1%)
Smoking status, n (%):			
- Never smoker	14 (46.7%)	14 (45.2%)	28 (29.2%)
- Former smoker	13 (43.3%)	16 (51.6%)	29 (30.2%)
- Current smoker	3 (10.0%)	0 (0.0%)	3 (3.1%)
Alcohol consumption, n (%):			
- None	22 (73.3%)	23 (74.2%)	45 (46.9%)
- Former consumer	1 (3.3%)	1 (3.2%)	2 (2.1%)
- Occasional	2 (6.7%)	6 (19.4%)	8 (8.3%)
- Chronic use	5 (16.7%)	1 (3.2%)	6 (6.2%)

**Table 2:** Intragroup comparisons of clinical parameters before and after the intervention, assessed using the Wilcoxon test.

Parameter	Group	Test Statistic	*p*-Value
Unstimulated saliva	Probiotic	0.0	0.0253
	Placebo	4.5	0.0339
Stimulated saliva	Probiotic	0.0	0.0016
	Placebo	12.0	0.7055
pH	Probiotic	13.0	0.0209
	Placebo	20.0	0.4054

**Table 3:** Intragroup comparisons of total and specific bacterial loads before and after the intervention, assessed by culture and qPCR methods using the Wilcoxon test.

Method	Bacterial Target	Group	Statistic	*p*-Value
Culture (log CFU/mL)	Total cultivable bacteria	Probiotic	147.5	0.0502
	*P. gingivalis*	Probiotic	43.0	0.1961
	*F. nucleatum*	Probiotic	99.0	0.0026
	*C. rectus*	Probiotic	87.0	0.5016
	Total cultivable bacteria	Placebo	172.0	0.2206
	*P. gingivalis*	Placebo	7.0	0.2367
	*F. nucleatum*	Placebo	184.0	0.3284
	*C. rectus*	Placebo	75.5	0.6627
q-PCR (log_10_ copies/mL)	Total bacterial load	Probiotic	131.0	0.0209
	*P. gingivalis*	Probiotic	37.0	0.1089
	*F. nucleatum*	Probiotic	115.0	0.0080
	*C. rectus*	Probiotic	109.0	0.8213
	*T. forsythia*	Probiotic	60.0	0.2668
	Total bacterial load	Placebo	201.5	0.5425
	*P. gingivalis*	Placebo	7.0	0.2367
	*F. nucleatum*	Placebo	194.0	0.4399
	*C. rectus*	Placebo	95.0	0.4761
	*T. forsythia*	Placebo	63.0	0.7960

Note: The Wilcoxon test was not applied to *Aggregatibacter actinomycetemcomitans*, as it was detected in only one of the 61 patients.

**Table 4:** Spearman correlation between q-PCR and culture methods for total and specific bacteria, by treatment group.

Bacterial Target	Group	Spearman r	*p*-Value	Interpretation
Total bacteria	Probiotic	0.886	3.32 × 10^−11^	Strong positive correlation
	Placebo	0.765	8.69 × 10^−7^	Strong positive correlation
*P. gingivalis*	Probiotic	0.997	2.20 × 10^−34^	Nearly perfect correlation
	Placebo	0.995	9.65 × 10^−30^	Nearly perfect correlation
*F. nucleatum*	Probiotic	0.923	1.60 × 10^−13^	Strong positive correlation
	Placebo	0.799	1.18 × 10^−7^	Strong positive correlation
*C. rectus*	Probiotic	0.870	2.06 × 10^−10^	Strong positive correlation
	Placebo	0.955	2.53 × 10^−16^	Strong positive correlation

## Data Availability

The data presented in this study are available on request from the corresponding author. The data are not publicly available due to ethical and privacy restrictions.

## Supplementary Materials

The following supporting information can be downloaded at: https://www.mdpi.com/article/10.3390/cancers17152459/s1, Table S1: Primers, Probes, and Amplification Conditions for Quantitative Detection of Oral Bacteria by qPCR.

## Abbreviations

The following abbreviations are used in this manuscript:

HNC	Head and Neck Cancer
IMRT	Intensity-Modulated Radiotherapy
CFU	Colony Forming Unit
GCF	Gingival Crevicular Fluid
qPCR	Quantitative Polymerase Chain Reaction
RTF	Reduced Transport Fluid
FAA	Fastidious Anaerobic Agar
BHI	Brain Heart Infusion
DNA	Deoxyribonucleic Acid
PCR	Polymerase Chain Reaction
