# Supplementary material for: Effect of a Probiotic Combination on Clinical and Microbiological Oral Parameters in Head and Neck Cancer Patients: A Randomised Clinical Trial
Source: Cancers (Basel). 2025 Jul 25;17(15):2459. doi: 10.3390/cancers17152459 (PMC12346730; doi:10.3390/cancers17152459)
Supplement: Supplementary file 1 [file cancers-17-02459-s001.zip › cancers-3724310-supplementary.pdf]

Table S1: Primers, Probes, and Amplification Conditions for Quantitative Detection of Oral Bacteria by qPCR

Bacterial Target	Primer sequences	Specific probes	Temp (°C)
Total bacteria (16S rRNA gene)	F [5'to3'] TCCTACGGGAGGCAGCAGT R [5'to3'] GCACTACCAGGGTATCTAAYCCTGTT	[6FAM] CGTATTACCGCGGCTGCTGGCAC[TAM]	65°C
A. actinomycetemcomitans	F [5'to3'] GAACCTTACCTACTCTTGACATCCGAA R [5'to3'] TGCAGCACCTGTCTCAAAGC	[6FAM] AGAACTCAGAGATGGGTTTGTGCCTTAGGG[TAM]	67°C
P. gingivalis	F [5'to3'] GCGCTCAAC-GTTCAGCC R [5'to3'] CACGAATTCCGCCTGC	[6FAM] CACTGAACTCAAGCCCGGCAGTTTCAA[TAM]	65°C
C. rectus	F [5'to3'] TTTCGCAGCGTAAACTCCTTTTC R [5'to3'] CGCTTGCACCCTCCGTAT	[6FAM] TCCGTGCCAGCAGCCGC[TAM]	65°C
F. nucleatum	F [5'to3'] GCATTTATTGGGCGTAAAGC R [5'to3'] GGCATTCCTACAAATATCTACGAA	[6FAM] CTCTACACTTGTAGTTCCG [TAM]	60°C
T. forsythia	F [5'to3'] GGGTGAGTAACGCGTATGTAACCT R [5'to3'] ACCCATCCGCAACCAA-TAAA	[6FAM] CCCG-CAACAGAGGGATAACCCGG [TAM]	55°C
